# Effect of perch access on perching, health and production outcomes in commercial broiler breeder flocks

**DOI:** 10.1016/j.psj.2022.102160

**Published:** 2022-08-30

**Authors:** G. Vasdal, S.G. Gebhardt-Henrich, Fernanda Tahamtani, K.E. Kittelsen

**Affiliations:** ⁎ Norwegian Meat and Poultry Research Centre, Lorenveien 38, 0515 Oslo, Norway; † Center for Proper Housing: Poultry and Rabbits, Division of Animal Welfare, University of Bern, Switzerland

**Keywords:** broiler breeders, perching, keelbone, footpad, floor egg

## Abstract

There is a need for more knowledge about perch use in broiler breeders and the potential effects of perches on health and production outcomes. The aim of this study was to investigate the use of perches by commercial broiler breeders, effect of perch access on keel bone fractures (**KBF**), footpad dermatitis (**FPD**) and number of floor eggs. Two commercial breeder flocks (Ross 308) reared at the same facility were observed during the production period. Half of each flock was provided with 15 cm perch/bird and the other half had no perches. The perch group had two types of perches; a steel plate mounted on the hen feeder lines “feeder perch” (15 cm high) and elevated plastic perches (5 cm high). Perching by hens and roosters was recorded during the dark period by counting birds on each of the two perch types in 10 sections and in the corresponding patches on the control side at 25, 35, and 45 wk of age (**WOA**). FPD was scored in 100 random hens in each group at 30 WOA and end of lay, KBF was scored by postmortem in 100 random hens in each group at end of lay, and number of floor eggs (n) in each treatment was scored daily. More hens perched on the feeder perch with the steel plate mounted, compared to the feeder line without the steel plate, but this difference decreased with age (*P* < 0.0001). Within the perch treatment, more hens perched on the feeder lines compared to the plastic perches at all ages (*P* < 0.0001). When combining number of hens on the plastic and feeder perches, on average 6.7 birds perched per meter perch, which is full capacity given an average shoulder width of 15 cm/bird. Perch use among the roosters was low overall, but more roosters perched in the perch group compared to the control group at 35 WOA (*P* = 0.007). Between 47 and 53% of the hens had KBF at the end of the lay. At 30 WOA, birds housed with perches were more likely to have lower FPD. Perch treatment did not affect number of floor eggs. In conclusion, broiler breeder hens perch when the perches are sufficiently high and allow all birds to perch simultaneously, and access to perches may have positive effects on FPD.

## INTRODUCTION

Optimal animal welfare includes good health, positive emotions and meeting behavioral needs. One important behavioral need for laying hens is perching (e.g., Newberry et al., 2001), which is an antipredator behavior still strongly embedded in the birds. Scientific knowledge of the importance of perches for the welfare of laying hens has led to the requirement of 15 cm perch per laying hen in the EU (Council Directive 199/74/EC). Broiler breeders, which produce fertile eggs for the broiler industry, are the same species as the laying hen, and we need more knowledge about the motivation for broiler breeders to perch.

Previous studies in broiler breeder pullets found that the pullets started using the perches as early as 2 wk of age, and the perches were increasingly used with age (Vasdal et al., 2022a). The birds showed no preference for perch materials or perch height (35 cm or 95 cm), but Hubbard JA787 pullets perched more than Ross 308 pullets. In another study with commercial broiler breeder flocks (Ross 308, Hubbard JA787 and Ranger Gold) provided with a total of 33 m of perches of metal, wood or plastic, Vasdal et al. (2022b) found that perch use during the light period was consistent with age and hybrid, and the highest perch (15 cm) was used more than the 5 cm high perches. However, the average perch use was only 0.44 birds/m perch which is a capacity utilization of less than 10%, given a shoulder width of 15 cm/bird (Aviagen, 2018). Similar results are reported by Brandes et al. (2020) with 40% perch utilization by Ross308/708 and Ross Ranger and by Gebhardt-Henrich et al. (2017), who report that more broiler breeders (Ross 308) perched when each bird was offered 14 cm of perch compared to 5 or 10 cm, but perch use was never above 50%. Mens and van Emous (2022) observed perching in broiler breeders and found that most birds perched on the wooden slats, followed by the plastic perches (both were placed 50 cm above the litter). The fact that platforms where the birds cannot grip around the structure appears to be preferred over perches is interesting and warrants further investigation.

With regards to age, some studies report a decline in perching behavior with increasing age in broiler breeder flocks (Gebhardt-Henrich et al., 2017, 2018; Mens and van Emous, 2022), while others do not report a reduction with age (Brandes et al. 2020; Vasdal et al. 2022b). In general, lighter birds perch more than heavier birds both in laying hens (Kozak et al., 2016) and in broiler breeders (Gebhardt-Henrich et al., 2018; Vasdal et al., 2022b). As broiler breeders grow heavier with age, it is important to know if their perching behaviour changes throughout the production period. Futhermore, time of day will also affect the perching behaviour; there seems to be a drastic increase in perching during the dark period compared to the light period in broiler breeders (2.07 birds/m vs. 0.73 birds/m perch) (Brandes et al., 2020) as well as in laying hens (Campbell et al., 2016). In order to record perch use at its main usage, perching behavior must therefore be observed during the dark period.

Another aspect is the perching behavior of roosters. Previous studies have only reported on the perching behavior of breeder hens (Mens and van Emous, 2022) or have not specified if the observed perching behaviors include roosters (e.g., Gebhardt-Henrich et al., 2018; Brandes et al. 2020). As regulations generally provide each bird in the flock with a certain allocated perch length, the motivations for roosters to perch is valuable information. Thus, the perching behavior of roosters should be included and specified when assessing perching in flocks of broiler breeders.

There cannot be good animal welfare without good health, and we need to make sure that any additions in the animals environment do not have negative health consequences. Keel bone fractures (**KBF**) have been defined as fragmentation, shearing, or bending of the keel bone (Casey-Trott et al., 2015). KBF in commercial laying hen production systems is alarmingly high, reported higher than 80% in some studies (e.g., Toscano et al., 2020). The prevalence of KBF in broiler breeders is less known. Gebhardt-Henrich et al. (2018) reported that KBF were more common in the breeder hybrid that perched most, but this effect was not found in Ross 308 breeders in Gebhardt-Henrich et al. (2017). However, in the latter study, percentage of perching birds was much lower compared to the former. If we provide birds with attractive perches that results in more perching, we need to investigate the potential effects on their keelbone. Furthermore, several studies report a high prevalence of footpad dermatitis (**FPD**) in broiler breeders (Thøfner et al., 2019; van der Oever et al., 2021). Although no negative effects of perches on FPD have been reported, this needs to be investigated further in commercial breeder flocks with access to perches.

Another key aspect of commercial poultry production is the production outcomes, and amount of floor eggs. Floor eggs are an economic problem as they require manual collection and are often dirty, which results in increased bacteria on the egg shell, a lower hatchability and reduced chick quality (van den Brand et al., 2016). The number of floor eggs can increase in a flock if access to the nests are limited, either due to the physical layout of the production system or aggression at the nest (Riber, 2010). One study found an increase in number of floor eggs when aviary tiers were present, but not when perches were present (Gebhardt-Henrich et al. 2018), likely due to the aviary structures providing dark and attractive areas to lay eggs. An increase in floor eggs due to presence of perches would be negative and lead to reduced willingness to provide perches. The effects of perches on number of floor eggs in commercial breeder flocks must therefore be investigated.

The aim of this study was to investigate 1) use of perches by commercial broiler breeders during the production period when provided with 15 cm perch/bird 2) effect of perch access on keel bone fractures and footpad dermatitis and 3) effect of perch access on number of floor eggs.

## MATERIAL AND METHODS

### Study Design

Two commercial breeder flocks (Ross 308) from the same grandparent flock and reared at the same rearer facility were observed throughout the production period (16–60 wk of age [WOA]) from June 2021 to January 2022 on 2 different broiler breeder farms in Norway. Each flock was divided in 2 along the length of the house, where one half of the flock was provided with 15 cm perch/bird (*perch group*) and the other half had no perches (*control group*).

Because the study did not involve any adverse animal handling, experimental manipulations or invasive procedures, it was exempt from approval of animal use by the Norwegian Food Safety Authority (Norwegian Regulations on Use of Animals in Research, 2015).

### Animals and Housing

One flock consisted of 2,500 hens and 200 roosters, the other 7,500 hens and 550 roosters (Table 1). The roosters arrived at the farm at 17 WOA and the hens came one week later, at 18 WOA. The roosters and the hens were randomly placed by the farmer on each side of the house. Both houses were fully insulated with mechanical ventilation and concrete floor with wood shavings, and elevated nest boxes and slats (Relax colony nest, Big Dutchman). The dimensions of the house, slats and nest boxes in the two flocks can be seen in Table 1. The flocks were managed according to standardized practices according to the breeding companies and Norwegian regulation with regards to feed, water, ventilation, litter and lighting (KSL, 2020, Aviagen 2018). The birds were fed a standard commercial diet (Strand Unikorn, Verpestart Muesli/Felleskjøpet Gull Kromat Muesli). The hen feeding lines (Champion feed chain, Big Dutchman) were placed on top of the slats, while the rooster feeders (MalePan, Big Dutchman) were placed in the littered area along the walls. The drinker lines with nipple drinkers with cups (Big Dutchman/Roxell spart line) were placed on the slats. One flock was culled on farm at 53 WOA and the other was sent to the abattoir at 63 WOA.

**Table 1:** Details of the flocks and house lay out.

Flock	House length (m)	House width (m)	Slat width (m)	Slat length (m)	Nest width (m)	Width of littered area (m)	Hens placed per side (n)	Roosters placed per side (n)	Length plastic perch (m)	Length hen feeder perch (cm)	Total (m) perch length	Perch length pr bird (cm)
1	38.0	12.0	3.6	36.0	2.0	1.5	1,250	100	120	108	228	16.8
2	100	15.0	3.6	98.0	2.0	2.5	3,750	275	294	290	584	14.6

### Perches

**Figure 1:**
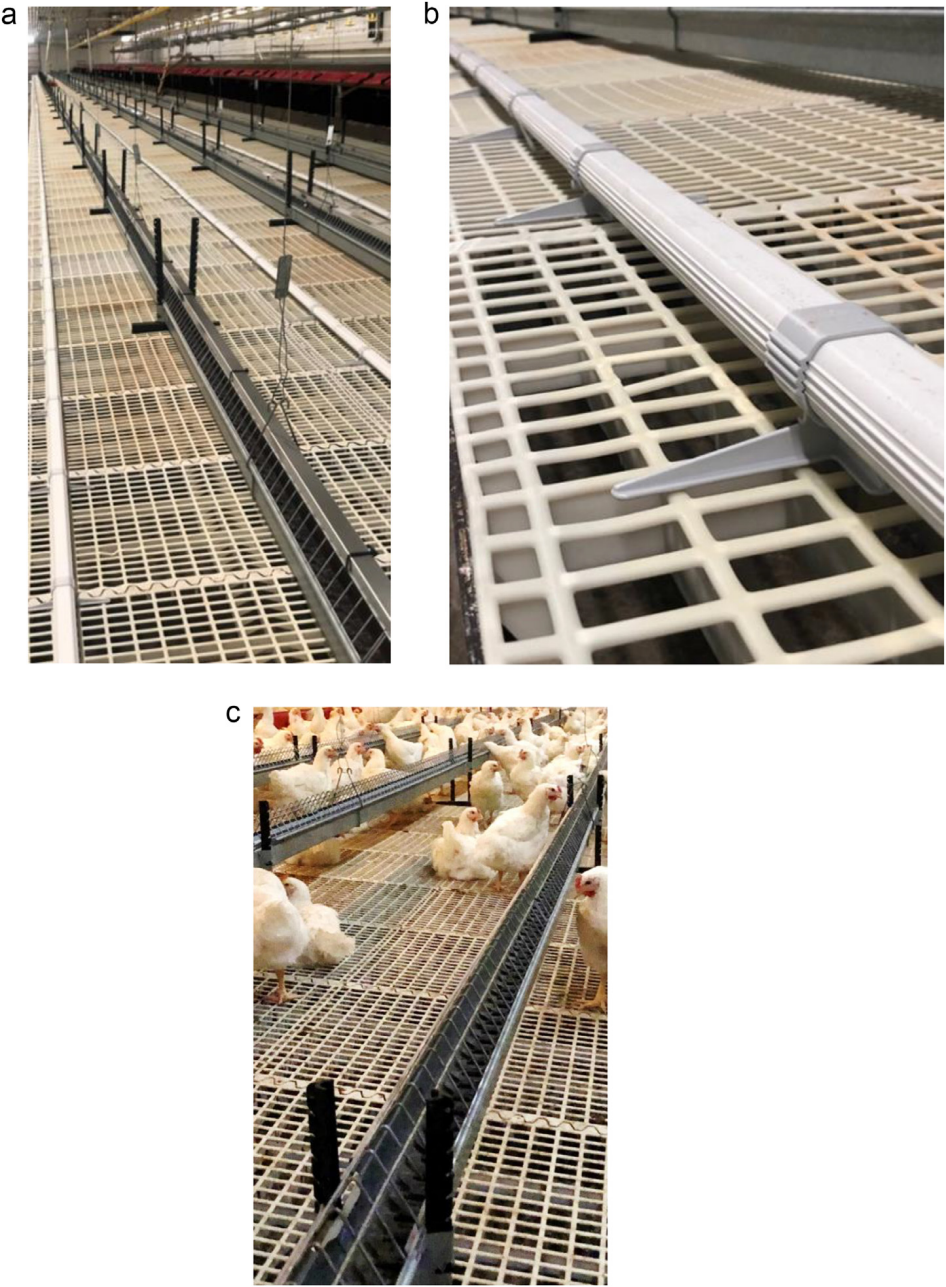
(A) The “feeder perch” with steel plates mounted on the hen feeder line. (B) The plastic perch on the slats (C) The hen feeder line without the steel plates on the control side.

The perch group in each flock was provided with 2 types of perches; metal perches on the hen feeder lines (“feeder perches”) and plastic perches. Both types of perches were placed on the elevated slats (350 cm wide, 60 cm high). The feeder perches consisted of steel plates placed on top of the hen feeder lines (45 cm high [Figure 1a]). The plastic perches (Ø 380 mm, APL/NAT, Big Dutchman) were placed between the feeder perches, in plugs on the slatted area (Figure 1b) (Big Dutchman, Plug PE APL) that elevated the perches 5 cm from the slats (Figure 1). Details of the length of each perch type and available perch space per bird is presented in Table 1. The perches were placed with 30 to 35 cm horizontal distance between them. There was 40 cm between the nearest perch to the drinker lines and 80 cm from the nearest perch to the entrance to the nest boxes. The control side also had the same hen feeder line, but without the steel plates on top of the feeder, thus the birds had to perch directly on the steel netting on the feeder line (Figure 1c).

Based on the average shoulder width of the Ross 308 hens and roosters (Aviagen, 2018), each meter of perch should theoretically accommodate a maximum of 7.1 (WOA 20 with 14 cm shoulder width), 6.6 (WOA 30 with 14.5/15 cm [hens/roosters] shoulder width) and 6.2 (WOA 40 with 15/16 cm [hens/roosters] shoulder width) birds respectively, if the birds perched shoulder to shoulder along the entire perch length.

### Observation of Perch Use

The slatted area along the length of the house was divided into 10 predefined sections (120 cm wide, 350 cm deep) on both the control side and perch side. Perch use by hens and roosters was recorded by direct observation 1 h after the lights were turned off by counting birds on the slats and on each of the 2 perch types in each section (P1–P10) and in the corresponding sections on the control side (C1–C10) at 25, 35, and 45 WOA. A head lamp (Hodelykt, oppladbar, Biltema) was used on the lowest intensity (1 lux) to avoid disturbance of resting birds.

### Health and Production Recordings

At 30 WOA and at end of lay (53/63 WOA), 2 observers visited the flocks and recorded footpad dermatitis (scale 0–4, Welfare Quality) in 100 random birds in each group. At end of lay, keel bone fractures (number of fractures via dissection post mortem) were recorded in 100 random hens in each group. The farmers kept daily recordings of floor eggs (n) on each side of the house. At the end of lay, we got the total number of dead birds per side from the farmer. Birds culled due to sorting were not included in the mortality.

### Statistical Analysis

Statistical analyses were performed using the software SAS 9.4 (SAS Institute Inc., Cary, NC). The number of hens observed perching per meter of perch available per observation section per flock per week of age was calculated. The number of hens perching on the feeder lines and on the plastic perches were analyzed using the mixed procedure. Fixed factors, where relevant, included treatment (i.e., perch or control), perch type (i.e., feeder line or plastic perch) and week of age, as well as their interactions. Observation section nested in flock was included in the model as a random factor. Perching behavior among the roosters was analyzed as the percentage of roosters in the observation section that were observed perching. This model was also carried out using the mixed procedure, with treatment, week of age and their interaction as fixed factors and observation section nested in flock as a random factor. Post-hoc analyses were performed with the Tukey test (Tukey's HSD test).

Due to the need to cull one of the flocks on farm at week 53 of age while the other was continued until week 64, the data on the health parameters at the end of lay could not be statistically analyzed. Therefore, only the data from the flocks at 30 wk of age were analyzed while the data from weeks 53 and 64 of age are presented as descriptive statistics. The data on footpad dermatitis at 30 wk of age were analyzed using a multinomial glimmix procedure with treatment as the fixed effect and flock as a random effect. The number of floor eggs per hen were analyzed using the mixed procedure with treatment, week of age and their interaction as fixed effects and the farm as a random effect. The mortality data are presented as descriptive statistics.

## RESULTS

### Use of Perches at Different Ages

**Figure 2:**
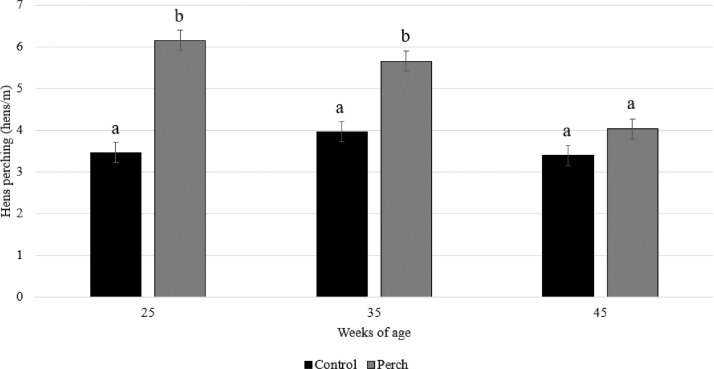
Number of hens per meter perch (LS means ± SE) perching on the feeder lines across treatments and weeks of age.

There was an interaction effect between the treatment and week of age on the use of the feeders as perches (F_2,95_ = 13.26; *P* < 0.0001). As can be seen in Figure 2, the number of hens per meter on the feeder perch was generally higher (5.3 ± 1.3 birds/m perch) in the perch group compared to number of birds on the feeder lines in the control group (3.6 ± 1.2 birds/m perch), but it decreased with age (*P* < 0.0001). As a result, more hens were seen perching on the feeder lines in the perch group than in the control group in weeks 25 (*P* < 0.0001) and 35 (*P* < 0.0001), but not in week 45 of age (*P* = 0.22). The number of hens per meter feeder line in the control group did not change with age (*P* > 0.05) (Figure 2).

**Figure 3:**
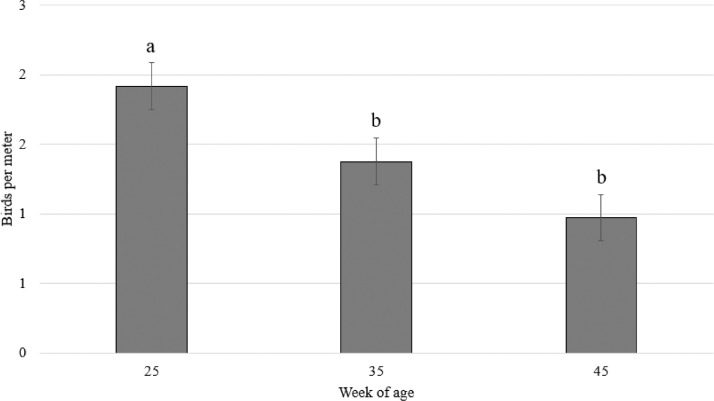
Number of hens per meter (LS means ± SE) perching on the plastic perches across weeks of age.

Regarding hen perching behavior on the plastic perches provided, there was an effect of age (F_2,38_ = 12.46; *P* < 0.0001; Figure 3). More hens where observed perching in these plastic perches on week 25 of age compared to week 35 (*P* = 0.02) and week 45 (*P* < 0.0001). There was no difference in perching behavior between weeks 35 and 45 (*P* = 0.1).

**Figure 4:**
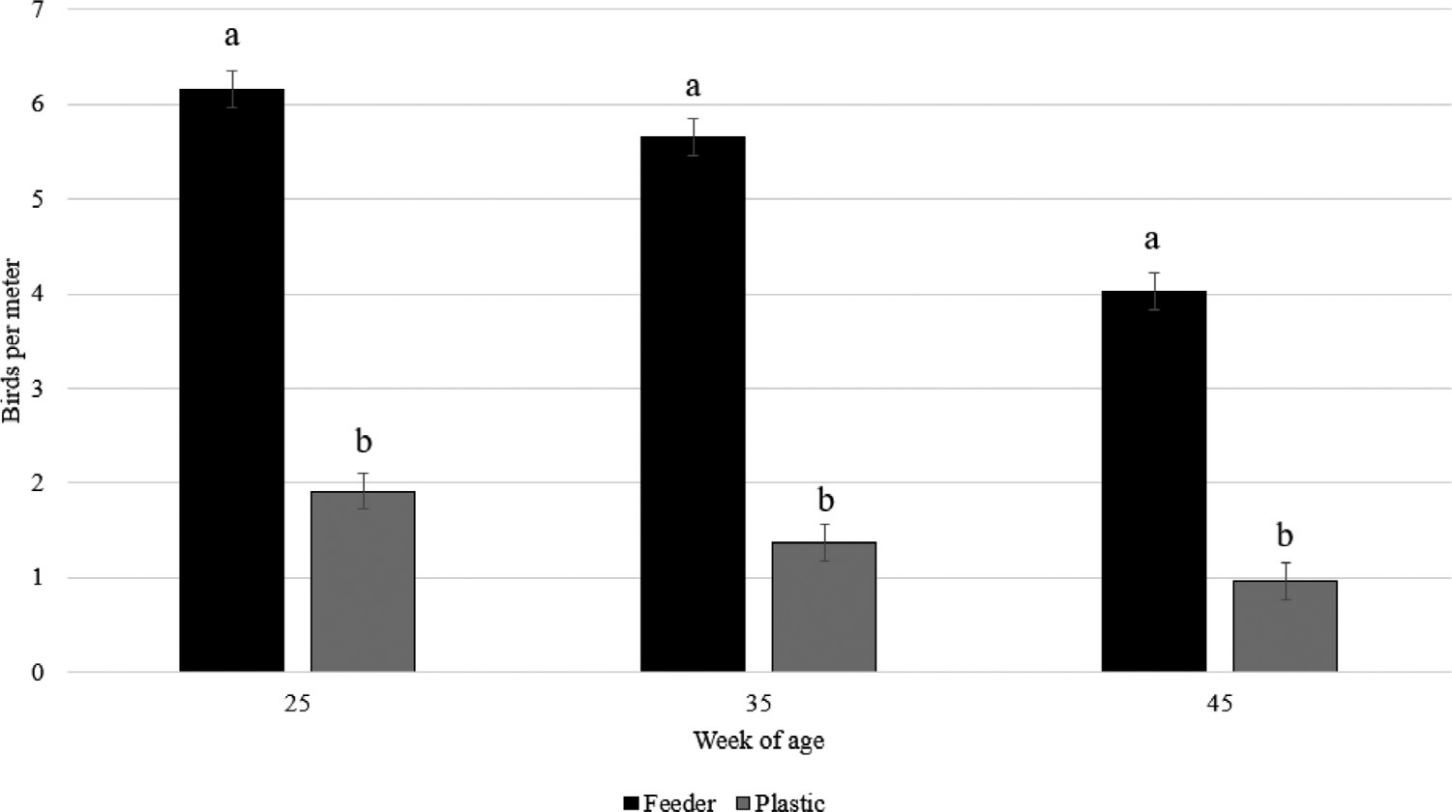
Number of hens per meter (LS means ± SE) perching on the plastic perches or on the feeder lines across weeks of age in the control group.

When comparing hen perching behavior on the feeder lines or on the plastic perches within the perch treatment, there was an interaction effect between type of perch and week of age (F_2,95_ = 9.74; *P* = 0.0001). As can be seen in Figure 4, more hens perched on the feeder lines compared to the plastic perches at all ages (P < 0.0001). Use of the feeder lines for perching declined from week 35 to 45 of age (*P* < 0.0001), and the use of the plastic perches was lower on week 45 compared to week 25 (*P* = 0.0007), but not compared to week 35 (*P* = 0.46).

### Rooster Perching Behavior

Perch use among the roosters was low overall (Table 2). There was, however, an effect of the interaction between treatment and week of age on the percentage of the roosters in each patch that were seen perching during the observations (F_2,100_ = 5.61; *P* = 0.005). More roosters were observed perching in the perch group compared to the control group at 35 wk of age (*P* = 0.007), but not at 25 wk (*P* = 0.70) or at 45 wk (P = 0.84).

**Table 2:** Mean number of roosters perching during the observations (birds/meter of perch).

Treatment	Week of age	N*	Mean	Std Dev	Minimum	Maximum
Control	25	20	0.00	0.00	0.00	0.00
Perch		20	0.07	0.10	0.00	0.28
Control	35	20	0.11	0.17	0.00	0.56
Perch		20	0.25	0.17	0.00	0.56
Control	45	20	0.13	0.19	0.00	0.56
Perch		20	0.05	0.08	0.00	0.28

⁎ N refers to 10 observation sections per treatment per flock.

### Keel Bone Fractures

In Flock 1, 51.5% of the birds in the perch group and 48.5% of the birds in the control group had fractures at 53 wk. In Flock 2, 53% of the birds in the perch group and 47% in the control group had fractures at 64 wk.

### Footpad Dermatitis

**Figure 5:**
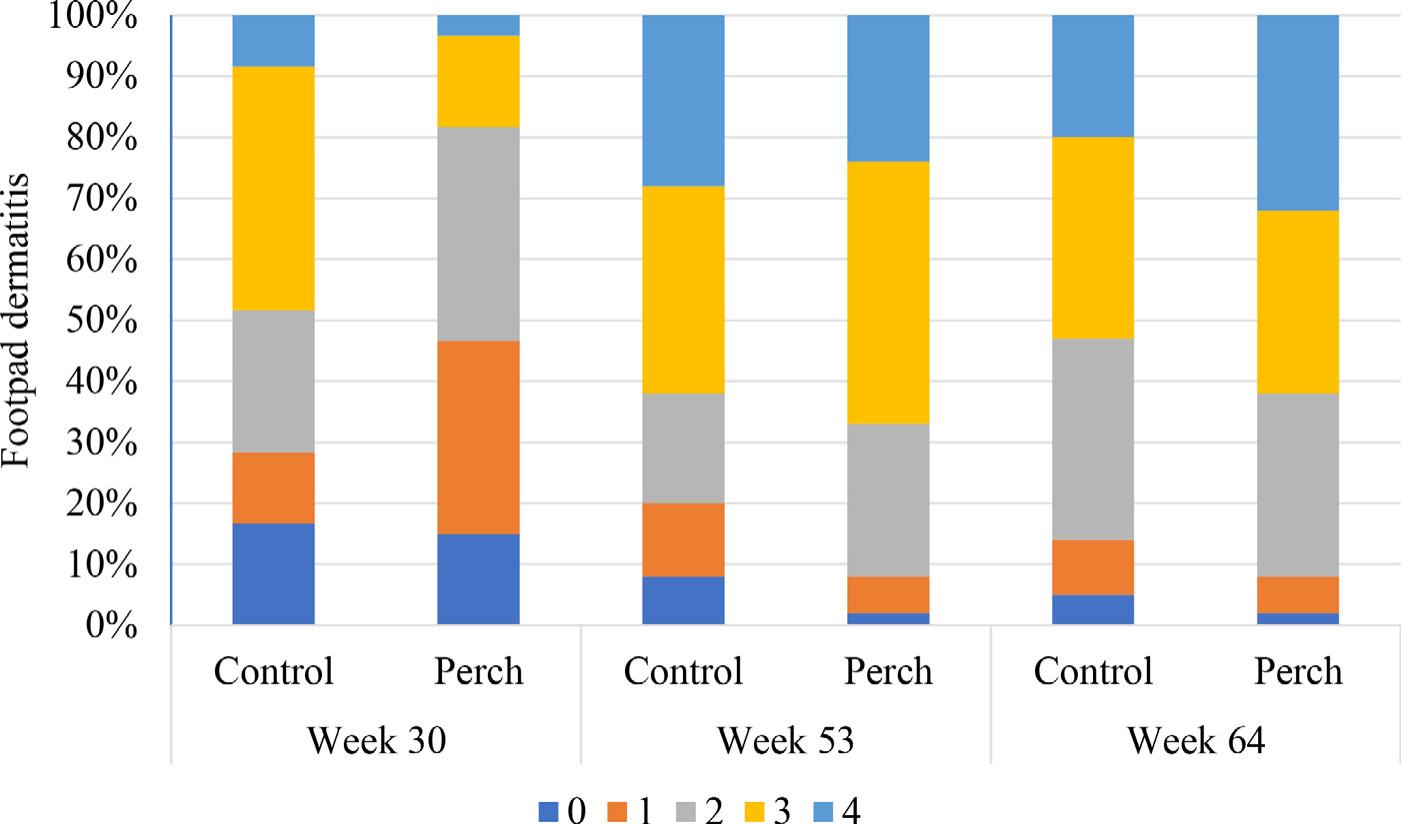
Frequency (%) of footpad dermatitis (FPD) across treatments and ages. Higher scores represent higher severity of the lesions. Numbers for week 30 included 2 flocks.

Scores for footpad dermatitis at each treatment per age is presented in Figure 5. At 30 wek of age, the birds housed with perches were more likely to have a lower score for footpad dermatitis (i.e., better footpad condition) compared to the control birds (F_1,115_ = 7.25; *P* = 0.008; odds ratio: 2.5).

Total mortality in the flocks ranged from 6.0 to 7.04% (Table 3). The number of floor eggs per group in each of the 2 flocks is presented in Table 3. There was no effect of treatment (F_1,55_ = 1.40; *P* = 0.24) or of the interaction between treatment and age (F_38,55_ = 0.23; *P* = 1.00) on the number of floor eggs. The number of floor eggs varied with age (F_38, 55_ = 7.01; *P* < 0.0001) as expected, with this number increasing during the first few weeks of the laying period, reaching its peak in week 27 (LS mean ± SE = 0.09 ± 0.02), and thereafter decreasing until the end of lay (the minimum was at week 57 = 0.02 ± 0.02).

**Table 3:** Total mortality (of total hens and roosters placed) and number of floor eggs per treatment in the 2 flocks.

	Total mortality (n)	Total mortality (%)	Floor eggs (total number)	Floor eggs (mean/day)
Flock 1^1^ – perch (birds placed n = 1,350	76	6.08	2321	11.9
Flock 1^1^ – control (birds placed n = 1,350	88	7.04	2009	10.3
Flock 2^2^ – perch (birds placed n = 4,025	240	6.0	3395	12.4
Flock 2^2^ – control (birds placed n = 4,025	284	7.0	3581	13.0

1 Daily data from 25 to 52 wk (195 d).

2 Daily data from 25 to 63 wk (274 d).

## DISCUSSION

The aim of this study was to investigate the overall use of perches by commercial broiler hens and roosters when provided with 15 cm perch/bird, and the potential effects of perch access on bird health and number of floor eggs. The results showed that more hens perched on the feeder lines when the feeders were fitted with steel plates on top compared to the hens who had to perch directly on the steel netting (average 5.3 hens vs. 3.6 hens/m perch). However, this effect decreased with age and by 45 wk of age, the number of hens perching on the feeder lines was similar between groups. A reduction in perching with age is reported in some previous studies (Gebhardt-Henrich et al., 2017, 2018; Mens and van Emous, 2022), while others report a consistent perch use throughout the production period (Brandes et al. 2020; Vasdal et al., 2022b). In the present study, the number of hens per meter of feeder line in the control group did not change with age and remained constant at around 3 to 4 hens/m perch. The difference between treatments in number of birds perching on the feeder is likely due to increased comfort added by the steel plates. This hypothesis can be further strengthened by behavior observations of perching birds, including duration of perching and number of balance movements (e.g., Pickel et al., 2010), which should be included in future studies. The reduction in number of perching hens in the perch group might be partly caused by increasing body weight, as lighter birds perch more than heavier birds both in laying hens (Kozak et al, 2016) and in broiler breeders (Gebhardt-Henrich et al., 2018; Vasdal et al., 2022a). The constant number of perching hens in the control group could represent the most motivated birds who prefer to perch, as the number of perching birds were similar by 45 wk of age.

When focusing on the plastic perches, fewer hens were observed on these compared to the feeder perches at all ages (average 1.4 birds vs. 5.3 birds/m perch), and the use of the plastic perch was reduced with age. Previous studies have found that the height of the perch is important (Brendler et al., 2014; Brandes et al., 2020; Vasdal et al., 2022b), and the present results clearly show that the breeder hens preferred the 15 cm high feeder perch compared to the 5 cm high plastic perch. In fact, more hens on the control side perched directly on the steel netting on the feeders (3.6 birds/m perch) than on the plastic perch. The plastic perch is mushroom shaped and could be considered more comfortable, but the birds did not prefer to perch on them. Thus, the perch must likely be of sufficient height to be used by broiler breeders.

The number of perching birds in both groups was higher than reported by previous studies. Given an average shoulder width of 15 cm/bird (Aviagen, 2018), each meter of perch can accomodate 6.6 birds. When combining the observed number of hens on the plastic and feeder perches, on average 6.7 birds perched per meter perch, which is full capacity (100% perch utilization). Previous papers report far lower numbers for broiler breeders: Vasdal et al (2022b): 0.44 birds/m perch; Brandes et al. (2020): 40% perch utilization and Gebhardt-Henrich et al. (2017): 50% perch utilization. One difference is that perching in the present study was observed during the dark period, when perching is known to be higher (Brandes et al., 2020). But even during the dark period, perching in Brandes et al. (2020) was still 2.07 birds/m perch. The use of perches by broiler breeders is likely affected by a combination of different factors, including bird factors such as age (Mens and van Emous, 2022), weight (Kozak et al., 2016), hybrid (Vasdal et al., 2022b) and early experience, in addition to location of the perches in the house (e.g., Vasdal et al., 2022b), the lay-out of the perch (e.g., Pickel et al., 2010) and the total amount of perch available (Gebhardt-Henrich et al. (2017). Poultry are prey animals and synchrony of behavior is an important anti-predator strategy. Thus, in order to stimulate a large proportion of the flock to perch, the perches must be comfortable, placed sufficiently high and allow all birds to perch simultaneously.

There has been little scientific focus on perching behavior in roosters and hardly any studies have reported perch use in roosters. Earlier studies on perching in broiler breeders tend to focus on the hens’ perching behaviour (e.g., Mens and van Emous, 2022), or do not differenciate between perching behaviour in hens and roosters (e.g., Brandes et al., 2020). In the present study, we found that perch use among the roosters was low, and only a handful of observations of perching roosters were made. More roosters were observed perching in the perch group compared to the control group at 35 wk of age, but the highest number of roosters observed perching was still only 0.25 roosters/m perch. The roosters in commercial breeder houses tend to spend their time in the littered area where their feed and water are located, while females tend to favor the raised slatted area (Leone and Estevez, 2008). A similar observation was made in the 2 flocks in the current study; most of the roosters were resting in the litter area after the light went out. From an evolutionary point, the roosters could be expected to have an innate motivation to rest on an elevated perch. There was avaliable space for the roosters to rest on the elevated slats if they had wanted to, but few roosters were observed on the slats.

There was no effect of perch treatment on the incidence of keel bone fractures (**KBF**) in any of the 2 flocks. On average, 50% of the investigated hens had KBF in the present study, which is alarmingly high. Although it is lower than the reported number for laying hens, it still represents a large welfare issue. In their study, Gebhardt-Henrich et al. (2017) reported that 24.75% of the breeders had moderatly to severely deformed keel bones, which included both fractures and deviations of the keel bone. However, this was assesed by palpation, which is known to underestimate the prevalence (Tracy et al., 2019). In contrast, we assessed KBF via dissection post mortem, which is considered the most reliable method. The lack of effect of perches on the prevalence of KBF is logical considering one of the hypotheses for KBF in laying hens is not related to physical factors in the house, but internal physiological factors such as the egg laying process (Eusemann et al., 2018, 2022) and early age for first egg, small birds and large eggs (Thøfner et al., 2021). However, the causes for KBF is likely multifactorial and future studies must focus on prevalence and potential causes of KBF in broiler breeders in a larger number of flocks and hybrids.

At 30 wk of age, the birds housed with perches were more likely to have better footpad condition compared to the control birds in both flocks, which is similar to results reported by Gebhardt-Henrich et al. (2017). This may be caused by reduced time spent in contact with the litter and slatted areas. In flock 1, that was culled at 53 wk of age, there was no difference between treatments with regards to footpad dermatitis (**FPD**). However, in the flock that was culled at 64 wk of age, the birds given access to perches tended to have higher severity of lesions compared to the birds in the control group. Some studies report a relationship between live weight and FPD, with heavier birds having more severe lesions (Gebhardt-Henrich et al., 2017), while other studies report no such relationships (Vasdal et al., 2022b). In the latter flock, live weight and litter quality was similar between sides. Thus, more studies are needed to investigate whether presence of perches indeed can affect the prevalence of FPD in breeders.

It is important to highlight that the present study investigated the conditions in only 2 flocks of broiler breeders, which is a very small sample size. Therefore, further studies including a more appropriate number of flocks is still needed to close these knowledge gaps. We found no effect of treatment on the number of floor eggs in the 2 flocks, which is in contrast to Gebhardt-Henrich et al. (2018) who found an increase in number of floor eggs when aviary tiers were present. The perches in the present study did not create dark areas that could be considered attractive areas to lay eggs, nor did they hinder the birds’ access to the nest boxes. The number of floor eggs varied with age as expected, with increasing numbers during the first few weeks of the laying period, reaching its peak in week 27 and thereafter decreasing until the end of lay. The effect of different perch layouts on floor eggs should be tested further.

In conclusion, more hens perched on the feeder lines when the feeders were fitted with steel plates on top, and the hens clearly preferred the higher feeder perch compared to the lower plastic perch. On average, 6.7 hens perched per meter of perch, which is full capacity given an average shoulder width of 15 cm/hen. Perch use among the roosters was low, and only a handful of observations of perching roosters was made. Around 50% of the hens had KBF, but prevalence of KBF was not affected by perch treatment. At 30 wk of age, the birds housed with perches were more likely to have better footpad condition but at 64 wk, one flock tended to have higher severity of lesions in the perch group. Perch treatment did not affect the number of floor eggs. Broiler breeder hens want to perch, and in order to stimulate a large proportion of the flock to perch, the perches must be comfortable, placed sufficiently high and allow all birds to perch simultaneously.

## Contributions

Guro Vasdal: conceptualizing, data sampling, writing. Sabine Gebhardt-Henrich: analyses, writing. Kathe Kittelsen: data sampling, writing. Fernanda Tahamtani: data sampling, analyses, writing.
